# Comparative Transcriptomic Analysis of the Larval and Adult Stages of *Taenia pisiformis*

**DOI:** 10.3390/genes10070507

**Published:** 2019-07-04

**Authors:** Shaohua Zhang

**Affiliations:** State Key Laboratory of Veterinary Etiological Biology, Key Laboratory of Veterinary Parasitology of Gansu Province, Lanzhou Veterinary Research Institute, Chinese Academy of Agricultural Sciences, Lanzhou 730046, China; zhangshaohua01@caas.cn; Tel.: +86-931-8342837

**Keywords:** *Taenia pisiformis*, de novo transcriptome, differential gene expression, development, reproduction

## Abstract

*Taenia pisiformis* is a tapeworm causing economic losses in the rabbit breeding industry worldwide. Due to the absence of genomic data, our knowledge on the developmental process of *T. pisiformis* is still inadequate. In this study, to better characterize differential and specific genes and pathways associated with the parasite developments, a comparative transcriptomic analysis of the larval stage (TpM) and the adult stage (TpA) of *T. pisiformis* was performed by Illumina RNA sequencing (RNA-seq) technology and de novo analysis. In total, 68,588 unigenes were assembled with an average length of 789 nucleotides (nt) and N50 of 1485 nt. Further, we identified 4093 differentially expressed genes (DEGs) in TpA versus TpM, of which 3186 DEGs were upregulated and 907 were downregulated. Gene Ontology (GO) and Kyoto Encyclopedia of Genes (KEGG) analyses revealed that most DEGs involved in metabolic processes and Wnt signaling pathway were much more active in the TpA stage. Quantitative real-time PCR (qPCR) validated that the expression levels of the selected 10 DEGs were consistent with those in RNA-seq, indicating that the transcriptomic data are reliable. The present study provides comparative transcriptomic data concerning two developmental stages of *T. pisiformis*, which will be of great value for future functional studies on the regulatory mechanisms behind adult worm pathogenesis and for developing drugs and vaccines against this important parasite.

## 1. Introduction

*Taenia pisiformis* (Cestoidea; Cyclophyllidea; Taeniidae) is a tapeworm with global distribution. The complete life cycle of *T. pisiformis* includes a larval stage that parasitizes the abdominal cavity of the intermediate hosts (lagomorphs) and an adult stage that inhabits the intestinal tract of the definitive host (canines) [1,2,3]. Infections in the definitive hosts occur when they ingest the internal organs of lagomorphs infected with *T. pisiformis* metacestodes, each of which can develop into an adult worm in the intestines of dogs. After worm maturation, eggs are released into the environment with the proglottids in the host’s feces. Lagomorphs become infected when they consume feed and water contaminated with the eggs of *T. pisiformis*. In China, *T. pisiformis* is a common parasite that infects rabbits. The larvae can cause severe health problems in rabbits, such as liver lesions, digestive disorders, and secondary bacterial infestation, resulting in economic losses in the rabbit breeding industry [4]. However, due to the absence of effective vaccines and deworming drugs, the parasitic disease is not currently well controlled. Therefore, identification of novel targets is urgently required to improve prevention of parasitic infections. 

For tapeworm-control strategies, vaccination and drug chemotherapies are considered effective ways to break the transmission cycle between the definitive host and the intermediate host. Dogs serve as the most common definitive hosts by harboring tapeworms in their intestines and play an active role in the long-term circulating transmission of tapeworms by shedding eggs into the environment [5,6]. Researchers have long focused on disrupting egg transmission from the source to control tapeworm infection. Specifically, a vaccine aimed at protecting dogs could directly break the transmission of eggs, which would be the most cost-beneficial measure for controlling tapeworm infections [7,8,9]. 

It is known that adult tapeworm can continuously generate new segments from the neck region behind the scolex once it inhabits the dog’s intestine. The reproductive systems develop within each segment, resulting in the production of numerous eggs. During the larva-to-adult metamorphosis, a unique property is the sharp strobilus development [10]. Therefore, it has been implied that numerous stage-dependent genes were activated in adult worms due to environmental factors, leading to the promotion of rapid growth and development of worm segments. However, the mechanisms involved in the fast development of *T. pisiformis* from larvae to adults and strobila movement, maturation and elongation, remain unclear.

High-throughput sequencing (RNA-seq) provides a powerful tool to analyze the parasite transcriptome to assist with transcripts identification and quantification [11,12,13]. In the present study, we performed the comparison of transcriptomic differences between the larval and adult stages of *T. pisiformis* using Illumina RNA-seq technology and de novo analysis. Subsequently, the transcriptomic data were validated by quantitative real-time PCR (qPCR). The findings will assist in identifying the differentially expressed genes (DEGs) and in exploring the molecular basis of biological processes involved in worm development, reproduction, and host interactions, as well as facilitating advanced investigations into crucial vaccine candidates and drug targets.

## 2. Materials and Methods 

### 2.1. Sample Collection

The larval *T. pisiformis* (TpM) were obtained from naturally infected rabbits at a local slaughterhouse in Zhengzhou, Henan Province, China. The freshly separated cysts from the peritoneal cavities were washed repeatedly in sterile phosphate-buffered saline (PBS, pH 7.4) to remove host components. The cysts were cultured in RPMI-1640 medium (Gibco, Grand Island, NY, USA) containing 1.25 µg/mL amphotericin B, 100 µg/mL streptomycin, and 100 units/mL penicillin for 12 h at 37 °C in an atmosphere of 5% CO_2_ to eliminate any bacterial contamination. Following another washing with PBS, cysts were placed into tubes (10 cysts/tube) and immediately frozen in liquid nitrogen for RNA extraction. The adult *T. pisiformis* (TpA) were obtained from experimentally infected dogs at our laboratory animal center. Dog infection with live TpM and TpA were performed as described by Toral-Bastida et al. [3]. The dogs infected with TpM were euthanized after 12 weeks, and three adult worms were recovered from the intestines of each animal, then treated as previously described [3]. The animal care and used protocol was approved by the Animal Ethics Committee of Lanzhou Veterinary Research Institute, Chinese Academy of Agricultural Sciences (No. LVRIAEC2016-006).

### 2.2. Illumina Hi-Seq Sequencing 

Total RNA was extracted from each sample using Trizol reagent (Invitrogen, Carlsbad, CA, USA) according to the manufacturer’s protocol. RNA purity and concentration were measured using the RNA Nano 6000 Assay Kit on the Agilent Bioanalyzer 2100 system (Agilent Technologies, Santa Clara, CA, USA) and Qubit^®^ RNA Assay Kit on a Qubit^®^2.0 Flurometer (Life Technologies, Carlsbad, CA, USA), respectively. RNA integrity was checked using a NanoPhotometer^®^ spectrophotometer (Implen, Westlake Village, CA, USA). Four sequencing libraries were constructed using the NEBNext^®^Ultra™ RNA Library Prep Kit for Illumina^®^ (New England Biolabs, Ipswich, MA, USA) following manufacturer’s recommendations. Briefly, messenger RNA (mRNA) was isolated from total RNA using magnetic beads with Oligo (dT) and cleaved into short fragments by mixing with the reaction buffer. Then, complementary DNA (cDNA) was synthesized using the fragmented mRNA as templates and random hexamers as the primer. Subsequently, the end repair, 3′ end adenylation, and adapters were performed. The fragments were purified with AMPure XP system (Beckman Coulter, Indianapolis, IN, USA), and the suitable fragments (150–200 bp in length) were chosen as templates for PCR amplification. The sample library quality was assessed on the Agilent Bioanalyzer 2100 system. The library preparations were sequenced on an Illumina HiSeq 2000 platform (Illumina, San Diego, CA, USA) by Biomarker Bioinformatics Technologies Co. Ltd. (Beijing, China), and paired-end reads (100 bp) were generated.

### 2.3. De novo Assembly, Expression, and Annotation Analysis

Prior to assembly, raw reads of fastq format were processed through in-house perl scripts. Clean reads were obtained by removing the adapter sequences, read containing poly-N (*N* > 10%), and low-quality reads from the raw reads. At the same time, Q20, Q30, GC-content, and the sequence duplication level of the clean reads were calculated. All high-quality clean data were combined together and de novo assembled using Trinity software (version: r20131110) [14] with the parameters as follows: min_kmer_cov 2, min_contig_length 200, group_pairs_distance 500, bflyHeapSpaceMax 20 G, bflyGCThreads 5, and seqType fq. Nonredundant unigenes were obtained with a length as long as possible. Clean reads were mapped back onto the assembled transcriptome, and all downstream analyses were based on the mapped reads. Finally, the nucleic acid sequences and amino acid sequences of the protein-coding region were predicted using TransDecoder software (version v2.0.1, http://transdecoder.sourceforge.net/). Unigene expression abundance was estimated by FPKM (fragments per kilobase of transcript per millions mapped reads) value [15] for each sample. Unigene function was annotated by BLASTx (https://blast.ncbi.nlm.nih.gov/, E-value < 10^−5^) based on the following seven databases: Nr (NCBI nonredundant protein sequences, ftp://ftp.ncbi.nih.gov/blast/db/), Swiss-Prot (a manually annotated and reviewed protein sequence database, http://www.uniprot.org/), GO (Gene Ontology, http://www.geneontology.org/), KOG (Clusters of euKaryotic Orthologous Groups, http://www.ncbi.nlm.nih.gov/KOG/), COG (Clusters of Orthologous Groups of proteins, http://www.ncbi.nlm.nih.gov/COG/), eggNOG (Orthologous groups of genes, http://eggnogdb.embl.de/), KEGG (Kyoto Encyclopedia of Genes and Genomes, http://www.genome.jp/kegg/), and Pfam (http://pfam.xfam.org/, HMMER E-value < 10^−10^). All raw data were deposited in the NCBI short read archive (SRA) database (http://www.ncbi.nlm.nih.gov/sra/) under the accession number: PRJNA506615.

### 2.4. Differentially Expressed Genes Identification between TpA and TpM 

Differential expression analysis of the two stages of *T. pisiformis* was implemented using the DESeq2 R package (version: v1.23.9) [16]. The resulting *p*-values were adjusted using the Benjamini and Hochberg’s approach for controlling the false discovery rate (FDR) [17]. In our analysis, an adjusted FDR value ≤ 0.01 and |log_2_FC (fold change)| > 1 were defined as the criteria of significantly differential expression. Next, a stringent threshold with FPKM > 1 in each TpA sample and FPKM = 0 in each TpM sample was set to identify the adult stage-specific genes of *T. pisiformis*, which were further compared with genes reported from a model cestode species, *Mesocestoides corti* [18]. 

To predict and classify the possible functions of the DEGs identified in TpA versus TpM, the GO enrichment analyses of DEGs were performed by the topGO R package (version1.10.0, http://www.bioconductor.org/packages/release/bioc/html/topGO.html) using Fisher’s test. KOBAS software [19] was also used to detect the statistical enrichment of DEGs in KEGG pathways. Values of *p* < 0.05 was considered statistically significant. In addition, to further find potential drug targets and vaccine candidates, the expression patterns of promising key DEGs and pathways contributing to the TpA growth and development were further investigated in this work. In particular, crucial genes involved in the transport and metabolism of nutrient substances and the Wnt signaling pathway were selected for analysis. The expression pattern analysis of the selected DEGs was performed using BMKCloud (www.biocloud.net). 

### 2.5. Quantitative Polymerase Chain Reaction (qPCR) Analysis

The qPCR assay was performed to validate the reliability and veracity of DEGs identified by the RNA-seq data. The total RNA from the two developmental stages of *T. pisiformis* was isolated as described above. qPCR was performed using the TransScript Green One-Step qRT-PCR Supermix kit (TransGen Biotech, Beijing, China) on an ABI 7500 Real-Time PCR Systems (Thermo Fisher Scientific, Waltham, MA, USA). Primers for the DEGs were designed using the OligoArchitect online tool (Sigma, St. Louis, MO, USA), and the detailed characteristics of the primers for all selected targets used in this study are summarized in Table S1. The PCR conditions were as follows: 45 °C for 5 min, 94 °C for 30 s, 45 cycles of 94 °C for 5 s, and 60 °C for 34 s. The melt curve of each amplicon was set as 95 °C for 15 s, 60 °C for 1 min, 95 °C for 30 s, and 60 °C for 30 s. *Tp-actin* gene (GenBank accession No. JX624787) was employed as an internal control in the assays [20]. The relative quantification of all selected genes was evaluated using the 2^−ΔΔ*C*T^ method [21]. Each experiment was performed in biological triplicates. A non-template negative control was included to confirm the lack of RNA contamination.

## 3. Results 

### 3.1. Sequencing and De Novo Transcriptome Assembly

**Figure 1:**
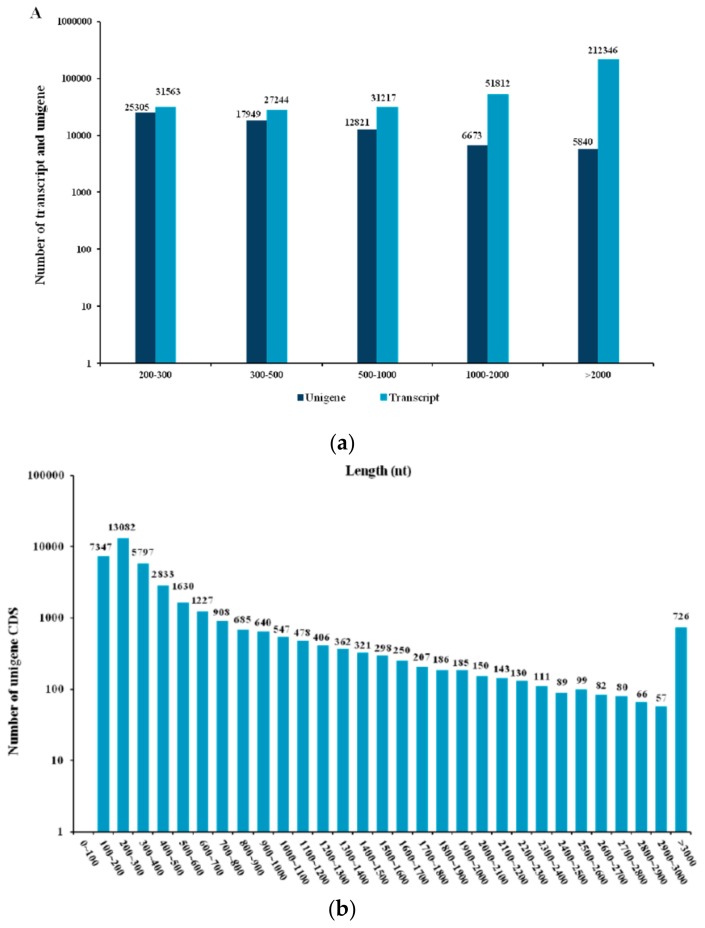
The size distribution of assembled unigenes of the *Taenia pisiformis* transcriptome: (**a**) Length distribution of transcripts and unigenes and (**b**) length distribution of unigenes coding sequences (CDS). Nt: Nucleotides

Four transcriptome libraries were constructed for Illumina sequencing from TpM and TpA. Due to the lack of a reference genome sequence for *T. pisiformis*, all raw reads were preprocessed for quality control. The clean reads were combined and de novo assembled into transcripts using Trinity software. As outlined in Table 1, 29.48 Gb of valid data were generated after quality filtration, containing 16.0 Gb and 13.48 Gb for the TpM and TpA libraries, respectively. The Q20 and Q30 of transcriptome library were 96.72% and 92.15%, respectively. Based on the clean data, 354,182 transcripts and 68,588 unigenes (>200 bp) were assembled de novo by the Trinity program from the libraries with average lengths of 3209 nt and 789 nt and N50 of 4893 nt and 1485 nt, respectively. Among these sequences, 12,821 unigenes (18.7%) were longer than 0.5 kb, 6673 unigenes (9.7%) had lengths longer than 1 kb, and 5840 unigenes (8.5%) were longer than 2 kb. Additionally, 39,122 coding sequences (CDS) were obtained from all assembled unigenes by TransDecoder software. The length distribution of the unigenes and CDS is shown in Figure 1. 

### 3.2. Gene Transcription Profile and Annotation 

Gene expression quantification showed that most paired-end reads were properly mapped back to the unigenes, and the mapping rates for TpM and TpA were 73.34% and 72.52%, respectively (Table S2), indicating that the transcriptome was well-assembled and that the unigenes were reliable for downstream study. To obtain additional insight into the assembled unigenes, BlastX comparisons (E-value < 10^−5^) were applied for a sequence similarity search against publicly available databases (Nr, GO, KOG, COG, KEGG, and SwissProt). As shown in Table 2, a total of 20,720 unigenes (30.21% of the total assembled unigenes) were annotated and had the highest match with 17,413 (25.39%) against the eggNOG database. In addition, 14,607 (21.29%) unigenes could be matched against the Nr database (Figure S1). In the Pfam, KOG, COG, Swissprot, GO, and KEGG database searches, 13,342 (19.45%), 10,920 (15.92%), 8613 (12.56%), 7794 (11.36%), 6149 (8.97%), and 6076 (8.86%) unigenes were functionally annotated, respectively. 

### 3.3. Identification and Classification of Differentially Expressed Genes 

**Figure 2:**
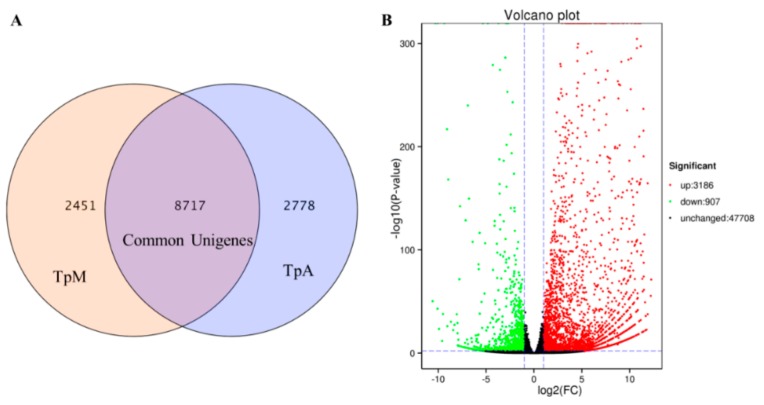
Distribution of expressed unigenes in the larval and adult stages of *T. pisiformis*: (**A**) The commonly or specifically expressed unigenes and (**B**) volcano plots of differentially expressed genes (DEGs) in TpA versus TpM. Unigenes with |log_2_FC| > 1 and adjusted *p*-value ≤ 0.01 were considered differentially expressed. Black splashes indicate genes with no significant change, and red and green splashes represent significantly upregulated and downregulated genes, respectively.

The FPKM analysis showed that 2778 and 2451 genes were expressed specifically in TpA and TpM libraries, respectively (Figure 2A). The significantly expressed DEGs were determined by setting threshold values of FDR ≤ 0.01 and |log_2_FC| > 1 in the pairwise comparison of TpA versus TpM. As a result, a total of 4093 genes (Table S3a) was differentially expressed in the two stages of *T. pisiformis*, comprising 3186 upregulated and 907 downregulated genes (Figure 2B). By comparing the results from *M. corti*, more stage-specific genes (495 genes) were found in TpA stage than that in adult *M. corti* (136 genes). The 495 genes contained 363 unannotated genes and 132 annotated genes with FPKM values ranging from 1.05 to 175.26. Then, 10 annotated genes were identified to be specific in two adult worms, including the enhancer of yellow 2 transcription factor, tubulin αchain, CCR4 NOT transcription complex subunit 2, calcium-binding protein, zinc finger protein, aquaporin 4, expressed conserved protein, and β-13-N-galactosyltransferase. Moreover, other stage-specific genes in TpA stage were identified, such as oncosphere antigen A, Thioredoxin fold, T box transcription factor TBX6, tetraspanin 1, Homeobox protein bagpipe, etc. The genes identified in the TpA stage and sharing consistency with *M. corti* are listed in Table S3b and Table S3c, respectively. 

**Figure 3:**
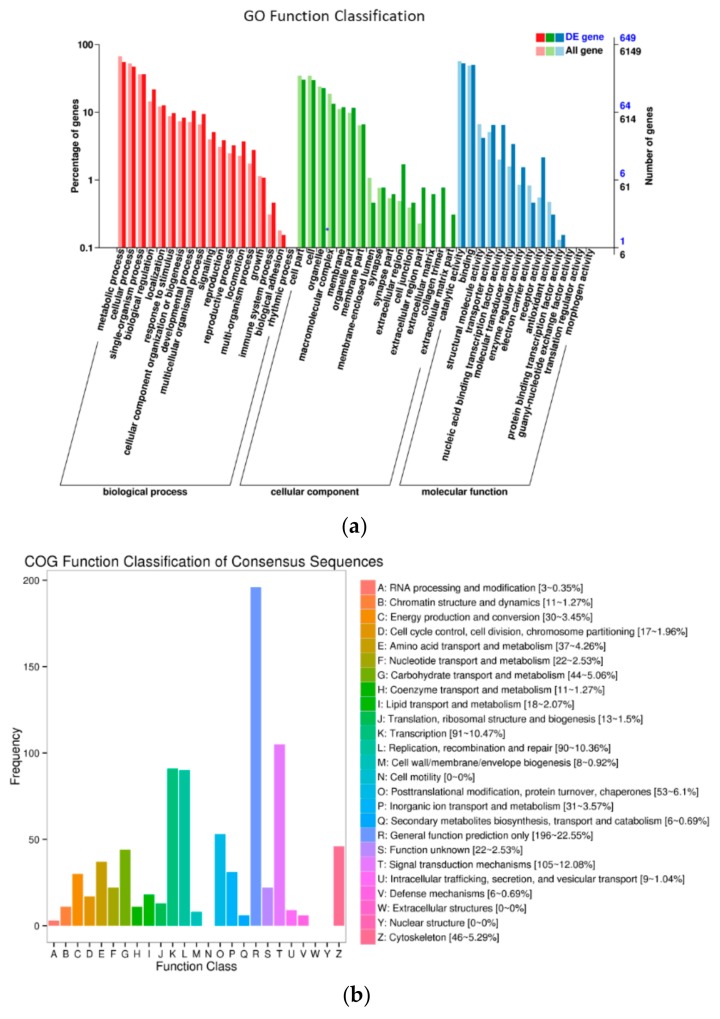
Functional annotation of DEGs of *T. pisiformis* transcriptomes in TpA versus TpM. (**a**) GO enrichment analysis of DEGs and (**b**) COG classifications of differentially expressed genes (DEGs).

**Figure 4:**
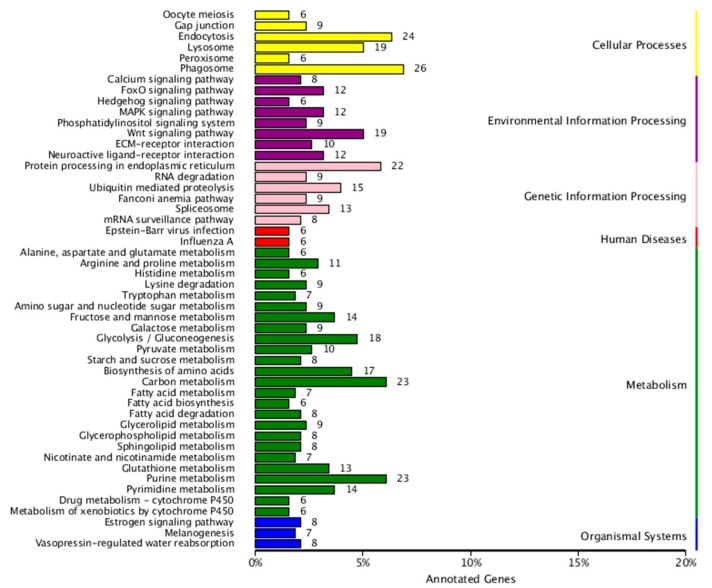
The enriched KEGG pathways of DEGs in TpA versus TpM: The numbers at the right of each column indicate the number of DEGs included in the corresponding pathway. The pathways were divided into six categories: “cellular processes”, “environmental information processes”, “genetic information processes”, “metabolism”, “human diseases,” and “organismal systems”. Criteria for statistical significance was *p*-value < 0.05.

Gene ontology functional analysis showed that 649 DEGs were enriched in 3 GO categories and 44 subcategories, including 219 GO terms with *p*-value < 0.05 (Table S4a,b). The most frequent GO classifications enriched in the biological process domain were “metabolic process,” “cellular process,” “single-organism process,” and “biological regulation”. In the cellular component category, the most highly enriched GO categories were “cell part,” “cell,” “organelle,” and “macromolecular complex”. In the molecular function category, DEGs were frequently involved in “catalytic activity,” “binding,” “nucleic acid binding transcription factor activity,” and “transporter activity” (Figure 3A). In addition, COG classifications showed that 869 DEGs were grouped into 22 functional categories (Figure 3B). The top clusters were as follows: “general function prediction only (196/869)”, “signal transduction mechanisms (105/869)”, transcription (91/869), “replication, recombination and repair (90/869)”, “posttranslational modification, protein turnover, chaperones (53/869),” and “cytoskeleton (46/869)”. KEGG enrichment revealed that 378 DEGs were specifically enriched in 206 pathways within the six main categories (Table S4c, Figure 4). Among these, 160 DEGs belonging to “metabolism” accounted for the highest proportion, followed by those relating to “environmental information processing” with 97 DEGs (Figure 4). With *p*-value < 0.05 as the threshold, the top 20 significant pathways of DEGs were enriched and listed in Table 3. Among these, “phagosome (26/378)” and “lysosome (19/378)” showed significant enrichment. Interestingly, several pathways related to worm development and reproduction were found, including the Wnt signaling pathway, MAPK signaling pathway and estrogen signaling pathway. The results indicated that more genes were active in the TpA stage and that the DEGs were associated mainly with changes in energy metabolism, gene transcription, and cell cycle in worm development.

### 3.4. Key Candidate Differentially Expressed Genes and Pathways Involved in TpA Development 

Differential expression analysis showed that most DEGs (3186/4093, upregulated) were significantly abundant in the adult stage and that the expression trend was consistent with previous studies (6910/10247, upregulated) described by Chen et al. [22]. In the current study, there were 230 DEGs (Table S5a) identified as being involved in the transport and metabolism of nutritive materials, including carbohydrate (72 unigenes), amino acid (46 unigenes), inorganic ion (44 unigenes), lipid (38 unigenes), and nucleotide (30 unigenes). Among significantly upregulated genes of interest, those DEGs related with the glycolysis/gluconeogenesis pathway, including hexokinase (HK), pyruvate kinase (PK), glyceraldehyde-3-phosphate dehydrogenase (GAPDH), enolase (ENO), fructose bisphosphate aldolase (FBA), phosphoglycerate mutase (PGM), and lactate dehydrogenase A (LDHA), were significantly upregulated with Log_2_FC values ranging from 4.85 to 11.32. In amino acid metabolism, the genes encoding cathepsin L cysteine proteinase, neutral amino acid transporter A, enteropeptidase, glutamate dehydrogenase (GDH), leucyl aminopeptidase (LAP), and arginase were highly expressed with Log_2_FC values from 5.28 to 9.13. Additionally, phospholipase D1, cytosolic fatty acid binding protein (FAB), SEC14-like protein, choline O acetyltransferase, and acetylcholinesterase were markedly upregulated with Log_2_FC values ≥ 2.26. The important upregulated DEGs in TpA versus TpM are listed in Table 4.

**Figure 5:**
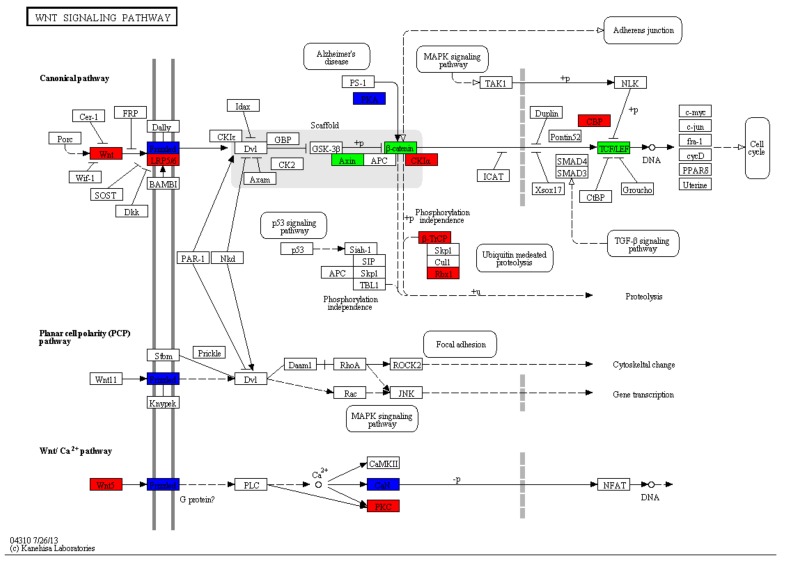
Unigenes involved in the Wnt signaling pathway: Red and green represent upregulated and downregulated genes, respectively. Blue indicates mix-regulated genes.

The Wnt signaling pathway was identified as an active biological pathway in adult worm development. The gene c29682.graph_c0 annotated with Wnt-04/Wnt-2/Wnt5 was upregulated with Log_2_FC values of 1.28. Further, the typical Wnt receptors frizzled 4 (Fz4) and low-density lipoprotein receptor-related protein (LRP5/6) [23], with Log_2_FC values of 5.96 and 1.11, respectively, were also found to be upregulated. Casein kinase Iδ (CKIδ), histone acetyltransferase p300 (HATs, CBP/p300), and cyclic adenosine monophosphate (cAMP) dependent protein kinase catalytic subunit (PKA) showed dramatically higher expression in the TpA stage with Log_2_FC values ≥ 10.12 (Figure 5). Moreover, some unigenes associated with worm reproduction were differentially expressed in the TpA group, of which Hsp90-like protein, sperm surface protein, and estradiol 17β-dehydrogenase were upregulated with Log_2_FC values of 6.01, 2.32, and 1.15, respectively. In the cell cycle process, several DEGs, such as cyclins, cyclin-B1-1, cyclin-B2-1, cyclin-B3, and cyclin cig2, were significantly expressed in the TpA stage with Log_2_FC values ≥ 2.61 (Table S5b), suggesting that cyclin B is highly active in the cell G2/mitotic-specific phases and plays an important role in the egg production of adult worms. 

### 3.5. Quantitative Polymerase Chain Reaction (qPCR) Verification

**Figure 6:**
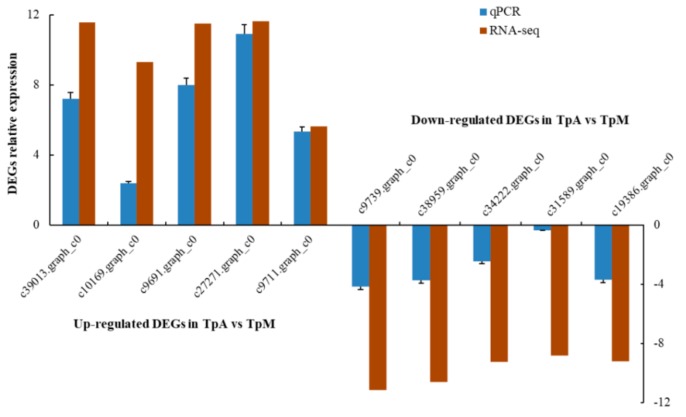
Validation of 10 selected DEGs derived from RNA-seq using quantitative PCR: *Tp-actin* gene was used as a reference gene. The error bars represent the standard deviation for three biological replicates.

To validate the RNA-seq results, the relative transcript levels of 10 DEGs genes (Table S1) were examined further by the qPCR assay with three biological replicates from each group. qPCR confirmed that the selected DEGs were successfully amplified with single bands in the expected sizes, and the gene expression patterns were in agreement with the results from RNA-seq analysis (Figure 6). These results illustrated the consistency of the results obtained from the assembled transcriptome from RNA-seq data and qPCR experiments.

## 4. Discussion

De novo transcriptome sequencing provides a powerful and cost-effective method for gene discovery in many organisms without a sequenced genome [14,24]. In recent years, comparative transcriptome analysis has been widely used to isolate and identify the differential expression of genes in helminth parasites, e.g., *Angiostrongylus* [25], *Fasciola gigantica* [13], and *Schistosoma* [11]. Likewise, the comparative transcriptional profile of *T. pisiformis* in different developmental stages is essential for understanding adult worm biology and for developing prevention strategies against this important parasite. In 2012, Yang et al. [26] performed the first transcriptome sequencing analysis for adult *T. pisiformis* and revealed the functional distribution characteristics and conserved genes sets in four cestode species, including *T. pisiformis*, *T. solium*, *Echinococcus granulosus*, and *E. multilocularis*. While the gene expression profiles from the different developmental stages of *T. pisiformis* were not conducted. More recently, Chen et al. [22] explored the difference of gene expression, metabolic pathway, and functional clustering in two development stages of *T. pisiformis*. These studies provided the extensive transcriptome dataset and valuable information in the understanding of gene expression profiles and functions of *T. pisiformis*. 

In the present study, we further performed a comparative gene transcriptional analysis between the larval and adult stages of *T. pisiformis*. By de novo assembly, a total of 29.48 Gb of clean sequencing data (average length >100 bp) were obtained and 30.2% (20,720/68,588) of the assembled unigenes were successfully annotated. At the same time, 69.8% of these unigenes were not annotated, which might be expressed uniquely in *T. pisiformis* and lack of gene homologues or orthologous groups in other taxa in the NCBI database. Compared with a similar study on *T. pisiformis*, the number of unigenes identified in this study (68,588 unigenes) was 1.9-fold greater than that reported in Chen’s study (36,951 unigenes) [22] but was similar to that described in Yang’s study (72,957 unigenes) [26]. Moreover, the number of differential expressed genes (4093 DEGs) identified from two developmental stages of *T. pisiformis* was less than that identified in Chen’s study (10,247 DEGs). The results might be partially due to the setting of various thresholds, |Log_2_FC| >1 and FDR ≤ 0.01 used in our data analysis and *p* ≤ 0.05 in Chen’s study [22]. However, the expression profiles of DEGs were consistent with Chen’s study of major upregulated genes expressed in adult stage of *T. pisiformis*. Regarding the stage-specific genes analysis, 132 annotated genes were presented in the TpA stage, such as calcium-binding protein, zinc finger protein, tubulin alpha chain, enhancer of yellow 2 transcription factor, oncosphere antigen A, Homeobox gene, etc. Some genes have been identified in previous studies of other cestode species. For instance, a stage-specific oncosphere antigen (TSOL8) with fibronectin type III domain has been confirmed as an effective vaccine molecule [27]. The genes coding tubulin isoforms, HoxB7, and zinc finger protein distinctively presented in adult worms of cestode species [18,28,29], indicating these genes mainly correlate with the process of cell differentiation and segmentation in adult stage. All of these results suggested that the substantial and high-quality transcriptome dataset of *T. pisiformis* was generated. Additionally, the GO and KEGG pathway analyses revealed that a large number of these candidate DEGs were associated with biological processes such as nutrition metabolism and worm development, suggesting that the segment development, maturation, and egg formation in the adult tapeworms might be controlled by numerous genes involved in diverse biological processes. Furthermore, the identified GO subterms and KEGG pathways could offer further information on the molecular basis of reproduction in *T. pisiformis*. Our findings facilitate exploring effective drug targets and vaccine candidates to suppress worm growth and egg production against *T. pisiformis* infection. 

During the life cycle of *T. pisiformis*, adult worms grow rapidly when consuming enough energy and nutrients to meet the requirements of metabolism, development, and egg production. Parasitic flatworms have lost the capability for de novo synthesis of certain nutrients, including fatty acids, sterols, as well as several amino acids [30,31,32]. The major sources of nutrition, including glucose, some amino acids, fatty acids and purine, are transported into the parasite for its requirements. In general, the adult worm achieves a higher level of gene transcript activity by accelerating metabolic pathways to meet the demands for the adaption of the parasitic lifestyle and survival of *T. pisiformis* in the host intestinal environment. It was demonstrated that glucose/glycogen is the main energy source for helminth parasites [33]. Our results indicated that the unigenes involved in the glycolysis/gluconeogenesis pathway were significantly more active in the TpA group, which was in line with the previous transcriptome analysis described by Yang et al. In addition to HK and PK, other important unigenes in catalyzed reactions, such as FBA, LDHA, ENO, PGM, GAPDH, and NADP-dependent malic enzym (Log_2_FC values: 10.79), were also significantly upregulated in the TpA stage. LDH is essential for converting the glycolytic product pyruvate to lactate under anaerobic conditions. NADP-dependent malic enzyme links the glycolytic and citrate cycle by the reversible oxidative decarboxylation of malate to pyruvate. Notably, some glycolytic enzymes can perform more complicated moonlighting functions in transcriptional regulation, cell apoptosis, and motility [34,35]. In tapeworms, FBA and ENO have been identified as multifunctional proteins participating in parasite motility and invasion [36,37]. Because of amino acid uptake in adult worm maturation, LAP was found to be significantly upregulated in the TpA stage. This observation was in accordance with our previous study, which found LAP to be expressed specifically in the TpA stage [20]. A prominent role of LAP enzyme is to perform proteolytic activities by digesting peptides or proteins [38,39]. Similarly, GDH provides nitrogen and glutamate (Glu) for maintaining energy homeostasis in parasites [40]. Cathepsin L cysteine proteinase from tapeworms can cleave macromolecules, such as IgG, fibronectin, and albumin [41,42,43]. These protease genes related to nutrition acquisition and pathogenesis were more active in the TpA group and, thus, of importance for segment development, motility, and invasion of adult worms. 

High fecundity is a striking biological feature in the TpA stage, during which adult worms in the dog intestine sexually produce eggs in each gravid proglottid. Some key factors are beneficial for tapeworm growth and maturation, such as exploiting fatty acids and cholesterol from host intestinal mucosa, as well as synthesizing steroid hormones (androgens and estrogens). In this respect, the tapeworms should be equipped with an efficient lipid binding system to uptake/transport host-derived lipids and express steroidogenic enzymes [44,45,46]. In the current work, the highly expressed lipid transporters, including FAB, phospholipase D1, and choline O acetyltransferase, played pertinent roles in capturing fatty acids from the host. Additionally, a range of functional unigenes closely associated with segmentation and sexual reproduction was upregulated in adult worms, including Hsp90-like protein, sperm surface protein, and estradiol 17β-dehydrogenase, suggesting that these genes may have played a pivotal part in strobilization, fertilization, and egg development in adult *T. pisiformis*.

The Wnt signaling pathway is an ancient and conserved signaling network that takes part in embryonic development, cell differentiation and proliferation, and the process of growth regulation [47,48]. In flatworms, the Wnt signaling pathway is responsible for regulating the formation of the anteroposterior (AP) axis, adult maturation, and/or egg production in the process of adult development in tapeworms [49,50]. To date, only five Wnt gene subtypes (Wnt1, Wnt2, Wnt4, Wnt5, and Wnt11) have been identified in flatworms. The expression analysis for all the Wnt subtypes in *Hymenolepis* showed that Wnt1 served as a segment polarity gene and was expressed only in adult worms, while Wnt2 was expressed only in larvae [51,52]. Wnt4 was involved in the canonical Wnt/β-catenin pathway and likely performed functions in the evolution of segmentation in platyhelminthes [53,54]. Wnt5 was shown to be involved in a traditionally noncanonical pathway (Wnt/Ca^2+^) and regulates specific β-catenin/coactivator interactions to promote cell differentiation in a PKC dependent manner [55]. The frizzled receptors participate in both canonical and noncanonical signaling and activate β-catenin/TCF transcriptional complexes [56,57]. The work of Dezaki and colleagues [50] demonstrated that eg-fz4 exhibited high expression levels at the three or more proglottid stage and the adult stage of *E. granulosus*. Our data also showed the presence of the same Wnt subfamilies, Wnt1, Wnt2, Wnt4, Wnt5, and Wnt11b, in *T. pisiformis*. Wnt1, Wnt4, Wnt5, Fz4, and LRP5/6 were dramatically upregulated in the TpA stage, indicating that the Wnt pathway is active in the adult development of *T. pisiformis* and likely associated with the formation of the anteroposterior (AP) axis, sexual maturation, and/or egg production in the adult worms. Moreover, some important molecules involved in Wnt/β-catenin signaling were markedly elevated in the adult stage, including CBP/p300 (Log_2_FC values: 10.12), PKA (Log_2_FC values: 10.79), and CKIδ (Log_2_FC values: 10.85), which interact with numerous transcription factors and increase target gene expression in the cell cycle [58]. Overall, the results from the current study indicated that both canonical and noncanonical Wnt signaling pathways are implicated in the transcriptional process of regulating TpA development. However, further analysis of the complex Wnt/FZD signaling system will be necessary to validate the function of Wnt genes in the regulation of stem cell maintenance and differentiation in *T. pisiformis*. 

Due to the lack of genomic information for *T. pisiformis* and missing gene homologues or orthologous groups in other taxa, some potentially novel genes associated with unique functions in the adult stage may remain undiscovered in the transcriptome of *T. pisiformis*. In the present study, although we could not determine which of the DEGs was the most important contributor to regulating TpA development, we did find important genes that could be therapeutic drug targets and vaccine candidates against *T. pisiformis* infection and possibly against cysticercosis. Secreted products derived from parasite were of value as new vaccine candidates against helminth infections [30]. The serine protease inhibitor, kunitz-type protease inhibitor, and LAP likely represent additional vaccine targets for *T. pisiformis*. At present, drug targets and their small molecule inhibitors are the primary focus in developing anti-parasite drugs. The parasite-derived enzymes [59], Wnt pathway [60], purine and pyrimidine pathways [61,62], are attractive chemotherapeutic targets for studying antiparasitic drugs. In addition, some potential drug target candidates were previously identified from several tapeworm genomes, including G-protein-coupled receptors, protein kinases, ion channels, etc. [31,32]. Recent studies showed that mefloquine could significantly inhibit enolase activity in adult schistosomes as well as schistosomula [63]. The transport activity of TGTP1 is greatly decreased by treatment with cytochalasin B or phloretin [64]. Given the importance of DEGs in carbohydrate metabolism in the adult stage, the associated molecular components in the glycolysis/gluconeogenesis pathway might be an effective target for the development of novel anti-cestode therapeutics [65]. It is anticipated that the dataset generated in this study will provide a solid foundation for further investigations into the DEGs identified in the adult worm stage in order to design potential parasite-specific compounds and effective vaccines for therapeutic and preventive purposes in tapeworm research.

## 5. Conclusions

In this work, we compared the transcriptomic data of larval and adult *T. pisiformis* and identified a large number of candidate DEGs involved in various biological processes. A total of 4093 DEGs were identified in the two lifecycle stages. DEGs enriched in the adult stage were associated with nutrient acquisition, AP axis formation, and egg development. These data will provide a valuable resource for future studies of *T. pisiformis* gene discovery and whole genome sequencing efforts on this tapeworm. The characterization of the comparative transcriptomic data has hints for a better understanding of the biological and physiological mechanisms of development and reproduction in *T. pisiformis* and will facilitate the development of interventional agents and effective vaccines or drugs to control this disease.

## Figures and Tables

**Table 1:** Summary of statistics of illumine transcriptome sequencing and assembly for *T. pisiformis*.

Sample	TpM	TpA	TpM + TpA
clean reads	53,815,898	45,351,168	99,167,066
Total bases (Gb)	16.0	13.48	29.48
(G + C) %	49.58	49.66	49.62
Q20 of clean reads (%)	96.79	96.65	96.72
Q30 of clean reads (%)	92.26	92.04	92.15
Numbers of transcripts	/	/	354,182
Mean length (transcript)	/	/	3209
N50 length (transcript)	/	/	4893
Total unigene	/	/	68,588
Mean length (unigene)	/	/	789
N50 length (unigene)	/	/	1485

Q20, Q30: The error rates of sequencing nucleotides <1% or 0.1%; N50: the shortest sequence length at 50% of overall length of spliced transcripts or unigenes; TpM: Larval stage; TpA: Adult stage.

**Table 2:** Summary of the statistics for the annotation of unigenes of *T. pisiformis*.

Annotated Databases	Annotated Number of Unigenes	300 ≤ Length < 1000nt	Length ≥ 1000 nt
COG	8613	3118	2362
GO	6149	2100	2493
KEGG	6076	1828	3195
KOG	10,920	3292	4802
Pfam	13,342	4476	5334
Swissprot	7794	2327	4031
eggNOG	17,413	5800	5430
Nr	14,607	4562	7003
All	20,720	6846	7097

COG: Clusters of Orthologous Groups; GO: Gene Ontology; KEGG: Kyoto Encyclopedia of Genes and Genomes; KOG: Clusters of euKaryotic Orthologous Groups; Pfam: Homologous Protein family; eggNOG: Orthologous Groups of Genes; Nr: Non-redundant.

**Table 3:** Top 20 KEGG pathways significantly enriched by DEGs in TpA versus TpM.

KEGG pathway	Pathway ID	DEGs No.	*p*-value
ECM-receptor interaction	ko04512	10	8.61 × 10^−7^
Vasopressin-regulated water reabsorption	ko04962	8	2.15 × 10^−6^
Neuroactive ligand-receptor interaction	ko04080	12	9.20 × 10^−5^
Phagosome	ko04145	26	6.38 × 10^−4^
MAPK signaling pathway	ko04010	12	9.15 × 10^−4^
Sphingolipid metabolism	ko00600	8	9.92 × 10^−4^
Fructose and mannose metabolism	ko00051	14	1.08 × 10^−3^
Estrogen signaling pathway	ko04915	8	2.68 × 10^−3^
Fanconi anemia pathway	ko03460	9	3.32 × 10^−3^
Nicotinate and nicotinamide metabolism	ko00760	7	3.63 × 10^−3^
Lysosome	ko04142	19	4.17 × 10^−3^
Morphine addiction	ko05032	3	4.62 × 10^−3^
Folate biosynthesis	ko00790	5	5.99 × 10^−3^
Wnt signaling pathway	ko04310	19	5.99 × 10^−3^
Platelet activation	ko04611	4	6.61 × 10^−3^
Glutathione metabolism	ko00480	13	8.78 × 10^−3^
Fatty acid biosynthesis	ko00061	6	9.19 × 10^−3^
Gap junction	ko04540	9	1.00 × 10^−2^
Galactose metabolism	ko00052	10	1.00 × 10^−2^
Melanogenesis	ko04916	8	1.11 × 10^−2^

DEGs No.: Number of DEGs mapped into each KEGG pathway.

**Table 4:** List of significantly upregulated unigenes associated with the transport and metabolism of nutrient substances in adult stage of *T. pisiformis.*

Transcriptome gene ID	log_2_FC	FDR	Gene annotation
Carbohydrate transport and metabolism			
c18758.graph_c0	11.3187	1.19 × 10^−^^72^	Fructose 1,6 bisphosphate aldolase
c10141.graph_c0	11.0122	5.32 × 10^−1^^64^	Hexokinase
c39583.graph_c0	9.7607	1.55 × 10^−^^32^	Glycosyltransferase
c39382.graph_c0	8.1210	4.30 × 10^−1^^69^	Pyruvate kinase
c39314.graph_c0	6.8155	1.71 × 10^−^^245^	Enolase
c9826.graph_c0	6.4117	0	Glyceraldehyde-3-phosphate dehydrogenase
c29987.graph_c0	4.8445	7.00 × 10^−1^^38^	Phosphoglycerate mutase
c18823.graph_c0	2.5367	1.87 × 10^−247^	alpha glucosidase
Amino acid transport and metabolism			
c39047.graph_c0	9.1324	6.51 × 10^−^^283^	Cathepsin L cysteine proteinase
c31605.graph_c0	8.1481	0	Cytosolic carboxypeptidase protein 5
c37268.graph_c0	8.0330	1.63 × 10^−87^	Neutral amino acid transporter A
c38431.graph_c0	7.5075	1.58 × 10^−40^	Enteropeptidase
c38964.graph_c0	7.4278	0	Glutamate dehydrogenase
c39380.graph_c0	6.7885	1.18 × 10^−^^216^	Cytosolic non specific dipeptidase
c9617.graph_c0	5.9653	0	Excitatory amino acid transporter
c9711.graph_c0	5.7216	0	Leucyl aminopeptidase
c18022.graph_c0	5.2819	0	Arginase-2
c35080.graph_c0	4.1657	3.16 × 10^−^^195^	Aromatic amino acid decarboxylase
c31258.graph_c0	3.9168	1.57 × 10^−1^^95^	Cytosolic carboxypeptidase 1
c32085.graph_c0	3.2382	3.90 × 10^−1^^13^	Cationic amino acid transporter
c9897.graph_c0	2.9532	0	Aspartate aminotransferase
c24988.graph_c0	2.5384	3.29 × 10^−^^23^	L-asparaginase
Lipid transport and metabolism			
c36506.graph_c1	8.5344	1.20 × 10^−^^9^	2-acylglycerol O-acyltransferase 2-A
c36947.graph_c0	6.9948	1.91 × 10^−^^85^	Phospholipase D1
c33881.graph_c0	4.4314	1.95 × 10^−^^67^	Protein bicaudal C1
c38848.graph_c0	3.4956	1.95 × 10^−^^23^	Cytosolic fatty acid binding protein
c9852.graph_c0	3.3705	1.64 × 10^−^^204^	Elongation of very long chain fatty acids
c32099.graph_c0	3.2596	8.83 × 10^−^^152^	Clavesin-2
c19373.graph_c0	2.6551	3.73 × 10^−^^100^	SEC14-like protein
c35827.graph_c0	2.4747	1.79 × 10^−^^35^	Choline O acetyltransferase
c39590.graph_c0	2.2559	1.65 × 10^−^^26^	Acetylcholinesterase
c37723.graph_c0	2.2167	8.66 × 10^−^^98^	Phosphatidate phosphatase
Inorganic ion transport and metabolism			
c10169.graph_c0	8.9320	1.21 × 10^−^^33^	Major intrinsic protein
c32341.graph_c0	7.0015	2.85 × 10^−^^63^	Sodium/hydrogen exchanger family
c18882.graph_c0	5.3420	0	Alkaline phosphatase
c24171.graph_c0	4.9420	9.49 × 10^−^^50^	ZIP Zinc transporter
c35064.graph_c0	3.8998	2.39 × 10^−^^33^	Sodium/hydrogen exchanger family
c37766.graph_c0	3.5535	1.23 × 10^−^^7^	KCNQ voltage-gated potassium channel
Nucleotide transport and metabolism			
c23643.graph_c0	7.8158	2.29 × 10^−^^117^	Adenylate cyclase 9
c10172.graph_c0	5.0739	2.84 × 10^−^^71^	Thymidine kinase
c10055.graph_c0	4.9673	2.52 × 10^−^^7^	Purine nucleoside phosphorylase
c31659.graph_c0	4.1400	0	Thioredoxin domain containing protein 3
c35152.graph_c0	4.0682	0	tRNA-specific adenosine deaminase
c31658.graph_c0	2.9613	2.23 × 10^−1^^47^	Equilibrative nucleoside transporter 3

FDR: False discovery rate; FC: Fold change.

## Supplementary Materials

The following are available online at https://www.mdpi.com/2073-4425/10/7/507/s1, Figure S1: The unigenes annotated by BLASTx against NCBI Nr database. The species distribution of unigenes is shown as a percentage of the total annotated sequences in Nr database with E-value < 10^−5^; Table S1: The selected DEGs, IDs, and primer pairs for qPCR; Table S2: The mapped numbers and rates of clean data mapped to assembled transcriptome; Table S3a: Updated DEG analysis results using DESeq2; Table S3b: Stage-specific genes identified in adult *T. pisiformis*; Table S3c: Ten stage-specific genes overrepresented in adult stage of *T. pisiformis* and *M. corti*; Table S4a: GO function annotation of DEGs in TpA versus TpM groups; Table S4b: Significantly enriched GO terms (*p*-value < 0.05); Table S4c: KEGG pathway mapping of DEGs in TpA versus TpM groups; Table S5a: A list of 230 DEGs associated with the transport and metabolism of nutrient substances; Table S5b: DEGs involved in Wnt pathway and cell cycle based on GO annotation.
